# A Metamaterial Surface Avoiding Loss from the Radome for a Millimeter-Wave Signal-Sensing Array Antenna

**DOI:** 10.3390/s24031018

**Published:** 2024-02-05

**Authors:** Inyeol Moon, Woogon Kim, Yejune Seo, Sungtek Kahng

**Affiliations:** 1 Department of Information & Telecommunication Engineering, Incheon National University, Incheon 22012, Republic of Korea; iy-moon@nissha.com (I.M.); wgon1002@inu.ac.kr (W.K.); m.june@inu.ac.kr (Y.S.); 2 Global R&D Center, NISSHA Korea, Inc., 7F, 26, Hwangsaeul-ro 312beon-gil, Bundang-gu, Seongnam-si 13591, Gyeonggi-do, Republic of Korea

**Keywords:** array antenna, millimeter-wave, signal sensing, radar, radio sensor, metamaterial surface, antenna gain

## Abstract

Radar systems are a type of sensor that detects radio signals reflected from objects located a long distance from transmitters. For covering a longer range and a higher resolution in the operation of a radar, a high-frequency band and an array antenna are measures to take. Given a limited size to the antenna aperture in the front end of the radar, the choice of a millimeter-wave band leads to a denser layout for the array antenna and a higher antenna gain. Millimeter-wave signals tend to become attenuated faster by a larger loss of the covering material like the radome, implying this disadvantage offsets the advantage of high antenna directivity, compared to the C-band and X-band ones. As the radome is essential to the radar system to protect the array antenna from rain and dust, a metamaterial surface in the layer is suggested to meet multiple objectives. Firstly, the proposed electromagnetic structure is the protection layer for the source of radiation. Secondly, the metasurface does not disturb the millimeter-wave signal and makes its way through the cover layer to the air. This electromagnetically transparent surface transforms the phase distribution of the incident wave into the equal phase in the transmitted wave, resulting in an increased antenna gain. This is fabricated and assembled with the array antenna held in a 3D-printed jig with harnessing accessories. It is examined in view of S_21_ as the transfer coefficient between two ports of the VNA, having the antenna alone and with the metasurface. Additionally, the far-field test comes next to check the validity of the suggested structure and design. The bench test shows around a 7 dB increase in the transfer coefficient, and the anechoic chamber field test gives about a 5 dB improvement in antenna gain for a 24-band GHz array antenna.

## 1. Introduction

Autonomous cars have been developed and put in the test phase or running over designated segments of a road. Convenience and effective time management during the car trip are often of concern, but they are left behind by safety of the passengers and pedestrians. To guarantee safety when you are behind the wheel, the car should be equipped with anti-collision sensors, exemplified by radars. Long before electronic devices for collision avoidance were introduced to driverless cars, airplane makers mounted radars on the flying vehicles to sense obstacles ahead of them from a long distance, relying on sending radio signals to suspicious directions and catching the reflected waves, which tells the user the calculated direction, size, and velocity. Obviously, an antenna system as part of the radar belongs to the transmitter and receiver made up of the wireless circuitry, power supply, and signal processor as well. There is a wide selection of the kind of antenna, from microstrip patches to horns dependent on the allowed area of the layout, and demanded weight and power-handling capability [1]. For relaxed-use cases of radars, the conventional types of antennas and familiar frequency bands are good enough to adopt. However, for critical applications, such as monitoring presence of hostile objects from a long distance or fast-moving vehicles like a UAM in the path, antennas and frequency bands must be carefully chosen, or their shortcomings need to be mended for accurate sensing [2,3,4].

As the object to be detected becomes located farther from the radar and feels like something shrinking, resolution in radio sensing should be higher, which is related to the higher directivity of the antenna’s radiated wave. When vehicles are running faster on motorways, the required bandwidth for signal processing ought to be wider to handle more information at a time. These necessitate the use of a millimeter-wave band radio, well known for shorter wavelengths and wider bandwidth. As is usual with automotive radars and 5G mobile base stations, an array antenna is employed to generate a high-directional emanating wave as a smaller geometry of densely populated radiating elements owing to the shorter wavelengths of 24- or 28- or 77-GHz bands. The antennas are as small as laid out on the rooftop of a millimeter-wave chipset. Liu et al. used a chip to feed an array antenna for 5G mobile connectivity [2]. TI and Infinion have developed MMIC beamforming chips combined with array antennas for radars [3,4]. Small as they are, these high-frequency antennas can narrow the beam and increase directivity to overcome path loss. This is verifiable by the antennas being open to the anechoic environment. Different from this ideal situation, the beam from the antenna for RF sensing becomes disturbed and weak by a cover layer called radome, required for a radar in reality. System-wise, the degraded beam, for instance, reduces transferred power as the dielectric loss and reflection by the radome causes trouble in the signal traveling and insufficient return power [5,6,7]. So to speak, problems with the beam end up with a drop in both radar resolution and accuracy [8].

When the antenna confronts a radome in a radar system, electromagnetic approaches are needed to lower or get rid of the dielectric loss from the cover layer. This specific layer is seen as a superstrate, and it can be modified as a metamaterial surface to bring uncustomarily positive functions as in [9]. As stated by books and articles on metamaterials, the electromagnetic fields and waves that enter a medium will go through reflection or weakening or change in velocity, and these phenomena can be mitigated by manipulating constitutive parameters of the medium material [9,10,11]. Because the constitutive parameters such as permittivity are embedded in the velocity and refractive index, manipulating them is equivalent to manipulating the phase or phase distribution of the wave. If the radome is replaced by a metamaterial superstrate to adjust the phase of the incident wave to what is demanded for a certain phase of the refracted wave, the electromagnetic disturbance will be minimized. Datthanasombat et al. changed the diverging wavefronts into the parallel wavefronts in a direction by adding the phase distribution of a multi-layered superstrate to the incident wave [12]. Kaouach et al. increased the operational frequency to a Q-band accompanying a wide-band and present a layered medium of a transmitarray, leading to a high gain [13]. Similarly, Dussoptia et al. moved the frequency band by one notch to take the benefit out of the expanded bandwidth and enhanced gain from a physically small antenna [14]. Wang et al. effectively increased the radiation aperture for a broadened field of view by using the optical approach [15]. The phase profile of each of those metamaterial superstrates is obtained to transform that of their incoming wave for an increase in far-field directivity.

This paper suggests a way to make the incident wave not to be disturbed by the cover layer using a flat metamaterial. While other metamaterial surfaces handle a single radiating element or a few, an array antenna with plenty of radiating elements is given as the source of radiation to send millimeter-wave signals propagated toward the radome. Especially, others show the area of the source antenna is much smaller than that of the metasurface, which gives less burden to the design process due to having a relatively large aperture plane; however, the task now is heavy since the area of the metasurface—the same as the area of the source antenna—is interpreted as having a small aperture and lower degree of freedom. The phase-compensating metasurface was designed based on observing the information in the incident wave and calculating the required phase distribution, and implemented by forming the metalized pattern on the dielectric substrate quite close to the material for the radome, like PTFE. First of all, an array antenna of 8-by-16 elements is designed as the source antenna to operate at the 24-GHz band. The cove layer, eight centimeters above the source antenna, becomes the plane for the field from the antenna to touch, and the phase of this incident wave is used to calculate the required phase distribution. This is discretized into the phase map for the metasurface, and all the pixels of the surface are expressed as the shapes of the metal pattern on the dielectric material. The metasurface consists of three 4350B sheets spaced by the air layer. As the metasurface is designed not to degrade the antenna beam, the dielectric sheets with metalized patterned cells do not introduce loss despite the relative dielectric constant being very different from 1.0. This electromagnetic transparent surface makes the radiated field from the array antenna avoid the loss and go forward smoothly, which is verified by the bench test comprising the source antenna in a jig, and the metasurface in a jig and the VNA. This reveals that the antenna experiences the transfer coefficient increasing by 7 dB as going farther from just overcoming the loss by the radome. This is taken to the anechoic far-field facility, whose experiments reveal the design has the antenna gain improved by 5 dB while enduring errors in harnessing; this includes the twisting of the RF cable and alignment between the source antenna and the metasurface on the mechanically turned table for mechanical scanning. The details of the design, structures, and performances will be addressed in the following sections.

## 2. The Source Antenna and Its Metasurface

As for a radar, an antenna is one of the building blocks to transmit and receive wireless signals at the backbone frequency. To carry out radio sensing with accuracy and far-reaching capability, an array antenna is a must-use electromagnetic gadget, particularly for overcoming the fast path loss of millimeter-wave band signals.

**Figure 1:**
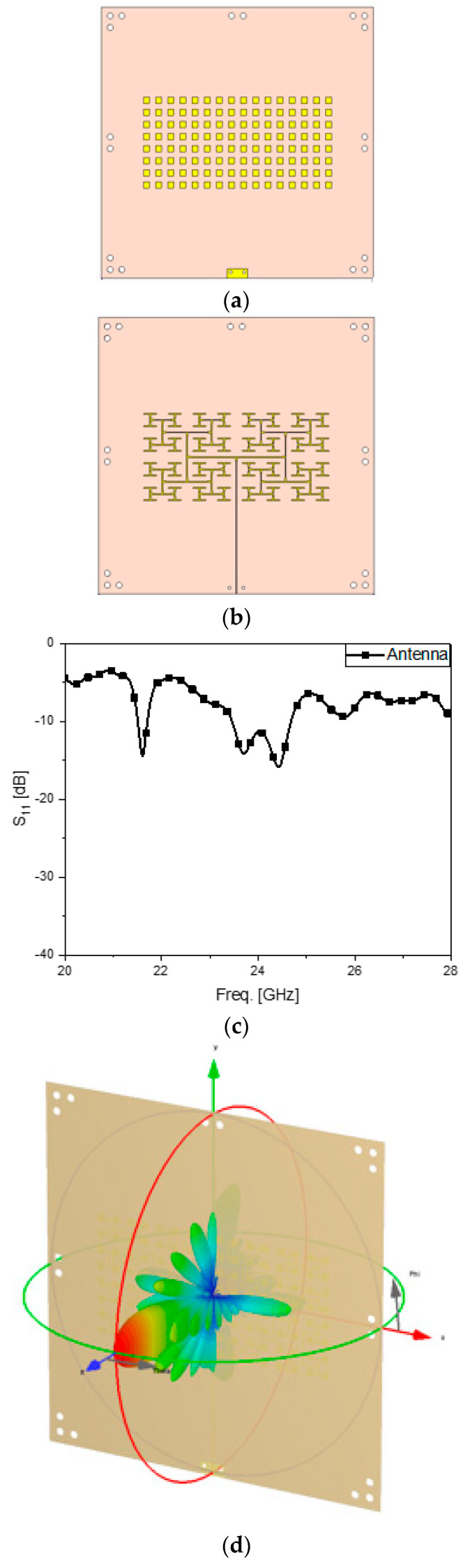

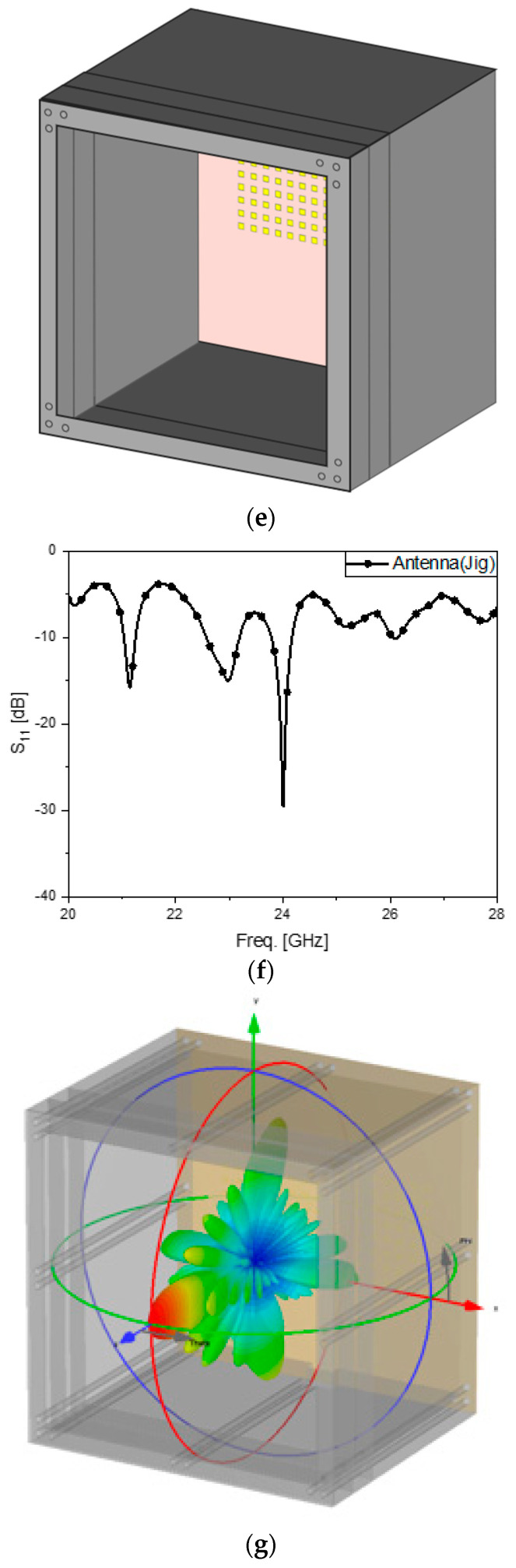
The array antenna in use. (**a**) Radiators on the front side; (**b**) feeder on the backside; (**c**) S_11_ as the reflection coefficient of the antenna; (**d**) far-field pattern of the antenna; (**e**) array antenna in a jig; (**f**) S_11_ as the reflection coefficient of the antenna in the jig; (**g**) far-field pattern of the antenna within the jig expressed in from red (strongest) through green (middle) to blue (weakest).

Figure 1 illustrates the array antenna as the source of radiation. It has 8 times 16 radiating elements equal to the area of 4.7 cm by 9.6 cm laid inside a 14 cm-by-14 cm dielectric substrate known as RT4350B. Each of the radiating elements is a 0.3 cm-by-0.3 cm rectangular patch resonating at 24.5 GH. The antenna of this size can make the antenna gain exceed 12 dBi. The periodic patches in Figure 1a are connected to the end points of the branches of the power divider presented in Figure 1b. The performance of the antenna is expressed by the reflection coefficient at the common port of the feed structure and the beam pattern in the electromagnetic simulation. S_11_ of Figure 1c shows the antenna has the resonance in the 24-GHz band, and the far-field pattern in Figure 1d shows us a high directivity leading to a gain of over 12 dBi, suitable for radar applications. With a view to imitating the realistic shape of a radar, the antenna is placed in a plastic mock-up as presented in Figure 1e. The open space in Figure 1a–d becomes closed by the four plastic walls, which affects the intrinsic field behaviors negatively at the millimeter-wave band. Multiple reflection of the minor lobes occurs due to the 24-GHz band signal being more sensitive to heterogeneous materials and stirs S_11_ rising by 8 dB and a frequency shift as in Figure 1f. Also, the far-field pattern becomes degraded with grown minor lobes, but largely the characteristics of the beam have not changed much, for the main lobe with a high antenna gain is still strong as observed in Figure 1g. The jig is introduced to hold the cover layer in front of the antenna. The radome should not be the conventional cover layer but must be capable of meeting several needs, like troubleshooting the degraded reflection coefficient and disturbed radiated wave. The cover structure is made as a metamaterial surface.

**Figure 2:**
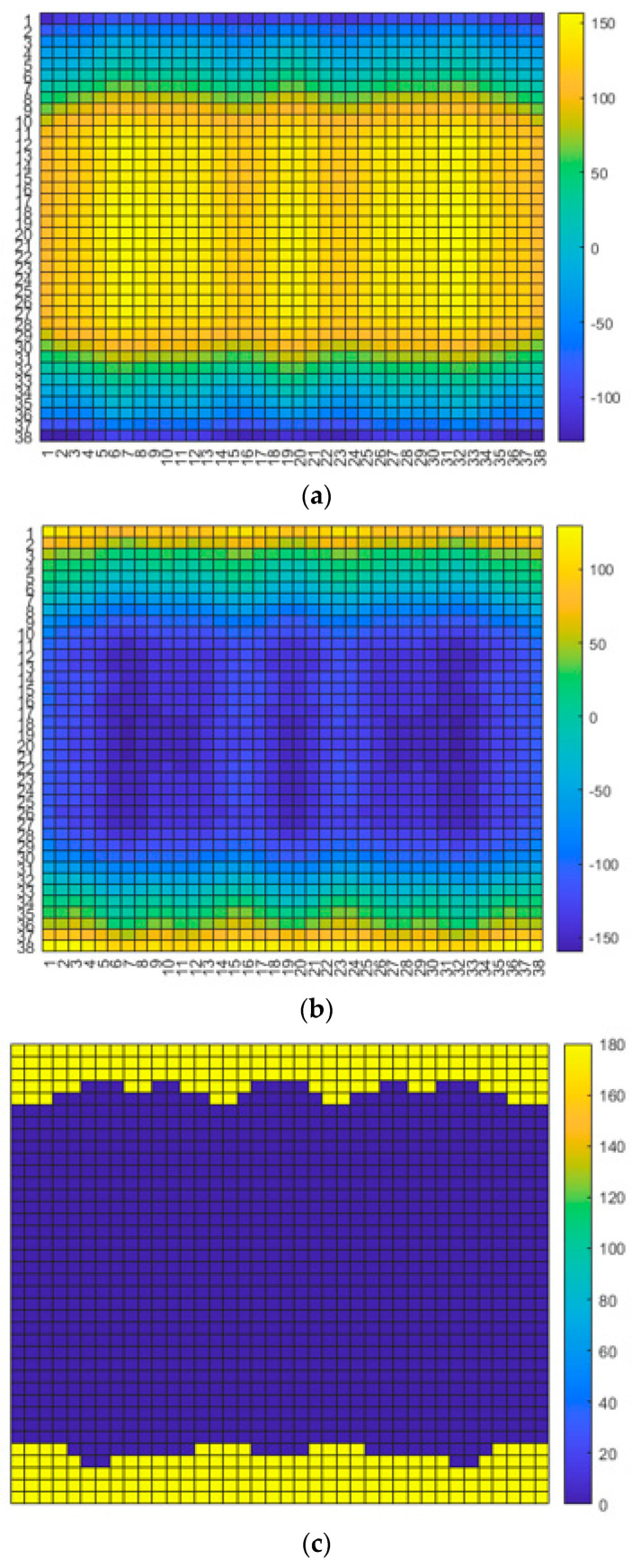

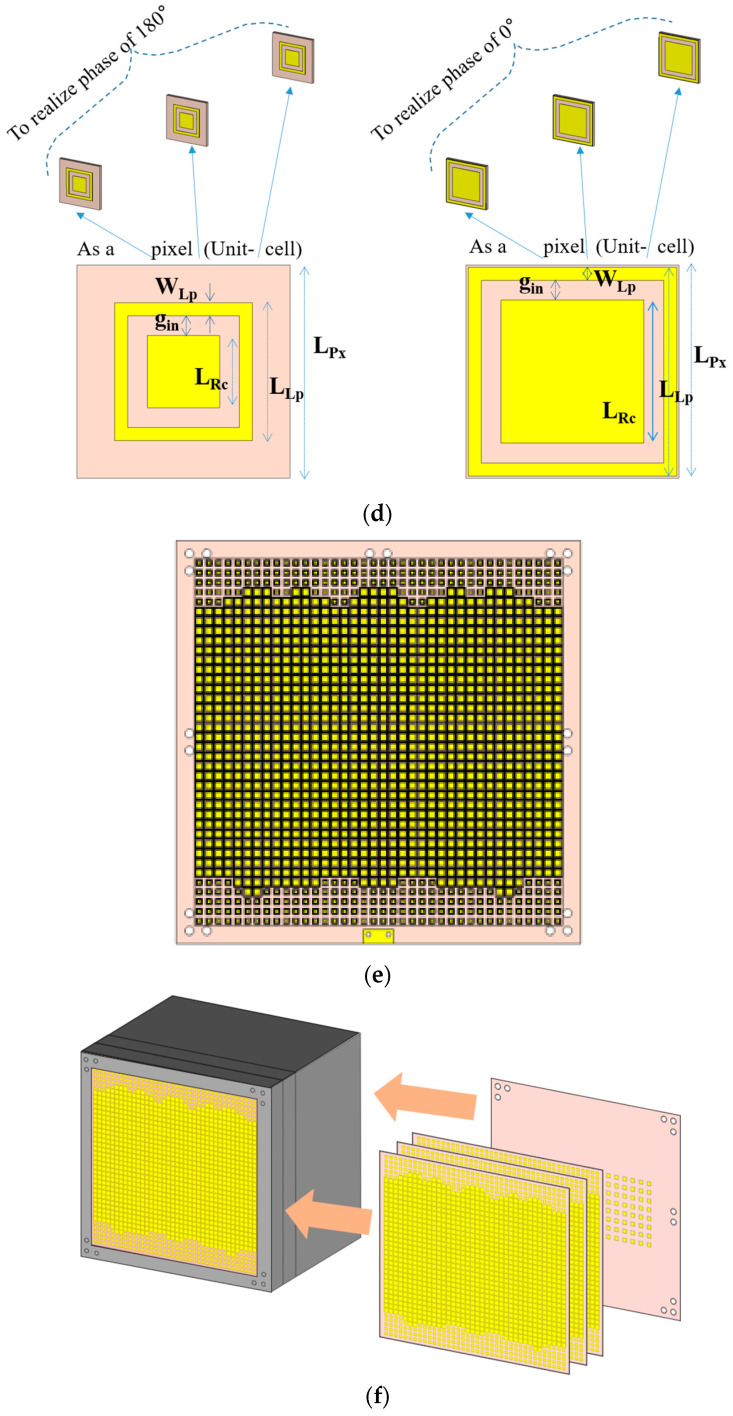

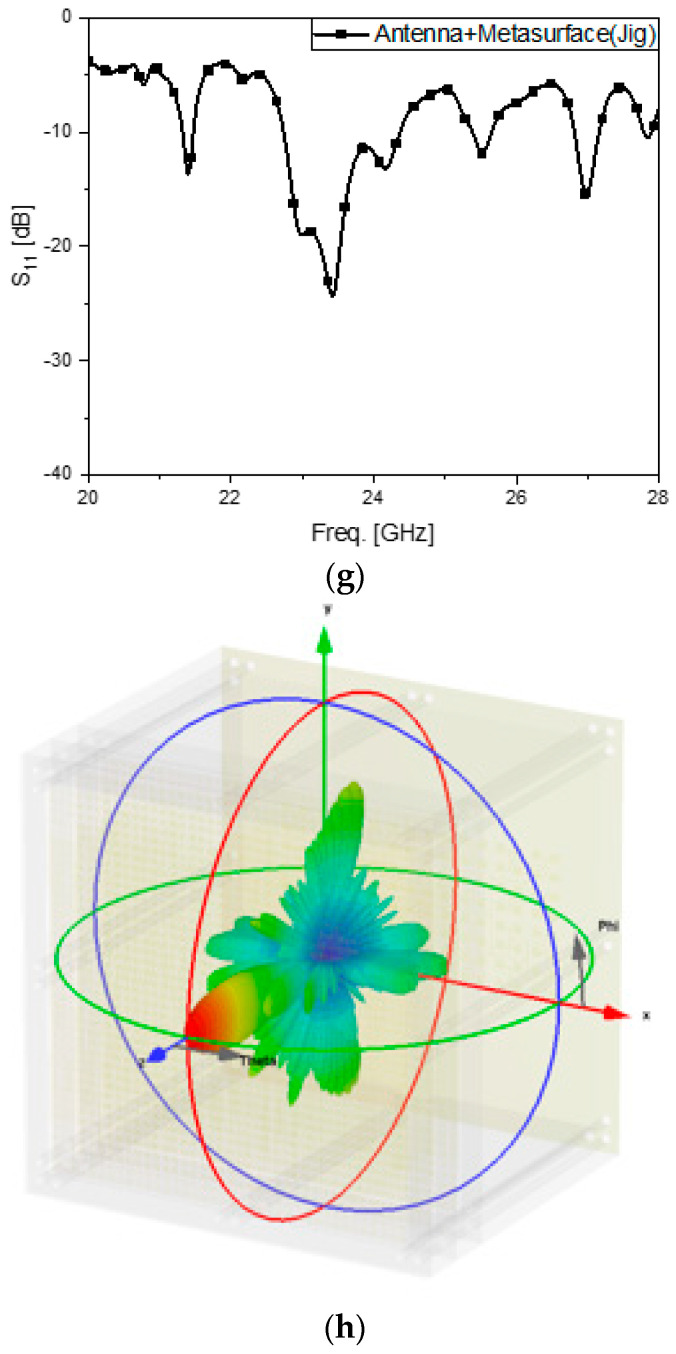
The metasurface for the array antenna in the jig. (**a**) Phase map of the incident wave; (**b**) the phase map required by the metasurface; (**c**) 1-bit expression of the phase map of the metasurface; (**d**) two types of pixels for the 1-bit phase map; (**e**) top view of the metasurface comprising the pixels; (**f**) bird’s eye view of the metasurface; (**g**) S_11_ of the metasurface-combined antenna in the jig; (**h**) far-field pattern of the metasurface antenna within the jig expressed in from red (strongest) through green (middle) to blue (weakest).

The design of the metasurface starts with the input information that is the phase distribution of the incident wave reaching the bottom plane of the cover layer from the array antenna, as plotted in Figure 2a. For the high directive radiated wave not to be interrupted, the refractive index is desired to be nearly zero, which makes the phase distribution almost equal and the beam directive continuous. This is made possible by the compensating phase map of the metasurface, as shown in Figure 2b. This complementary phase profile mathematical in Figure 2b is quantized to a 1-bit version, as shown in Figure 2c, in the course of physical realization. Phases of 180° and 0° by the 1-bit coding system painted in yellow and blue correspond to the left and right pixels of Figure 2d, respectively. The geometrical parameters of 180°- and 0°-pixels are given as follows.

As the geometry whose elements are in Table 1 comprising a metal square inside a metal loop for either pixel 180° or pixel 0° is stacked on the tops of two of its kind and spaced with the 1-cm thick air layers, the phase of each pixel is obtained. If a complicated metal shape and a much thicker substrate are adopted for a pixel, the number of its layers and volume will be diminished remarkably, but this ends up as pricy manufacturing. Using the pixels of Figure 2d and spreading them onto the entire plane, the metasurface becomes Figure 2e. The new cover layer is combined with the source antenna, as shown in Figure 2f. S_11_ of the overall structure is improved, as shown in Figure 1g compared to Figure 1f. As for the radiated field pattern, it is still very directive as the antenna without electromagnetic disturbance. The effect and frequency responses the design has pursued, along with the full-wave simulation, will be experimentally scrutinized in the following section.

## 3. Test Bench Examination, Anechoic Chamber Test, and the Analyses of the Results

The array antenna as the primary source and its metasurface were fabricated by using the PCB etching technique, and they were assembled in a mock-up enclosure made by 3D printing. The two specimens are shown in the photographs below.

**Figure 3:**
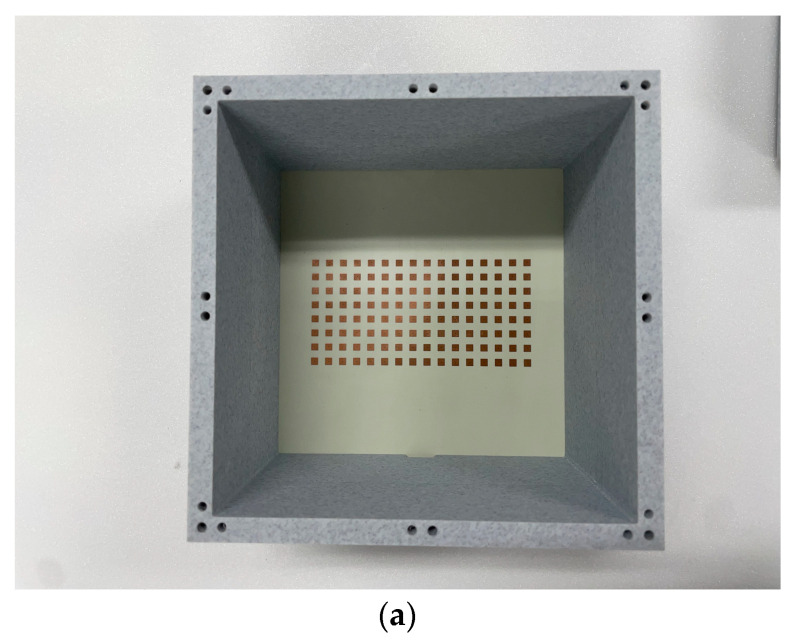

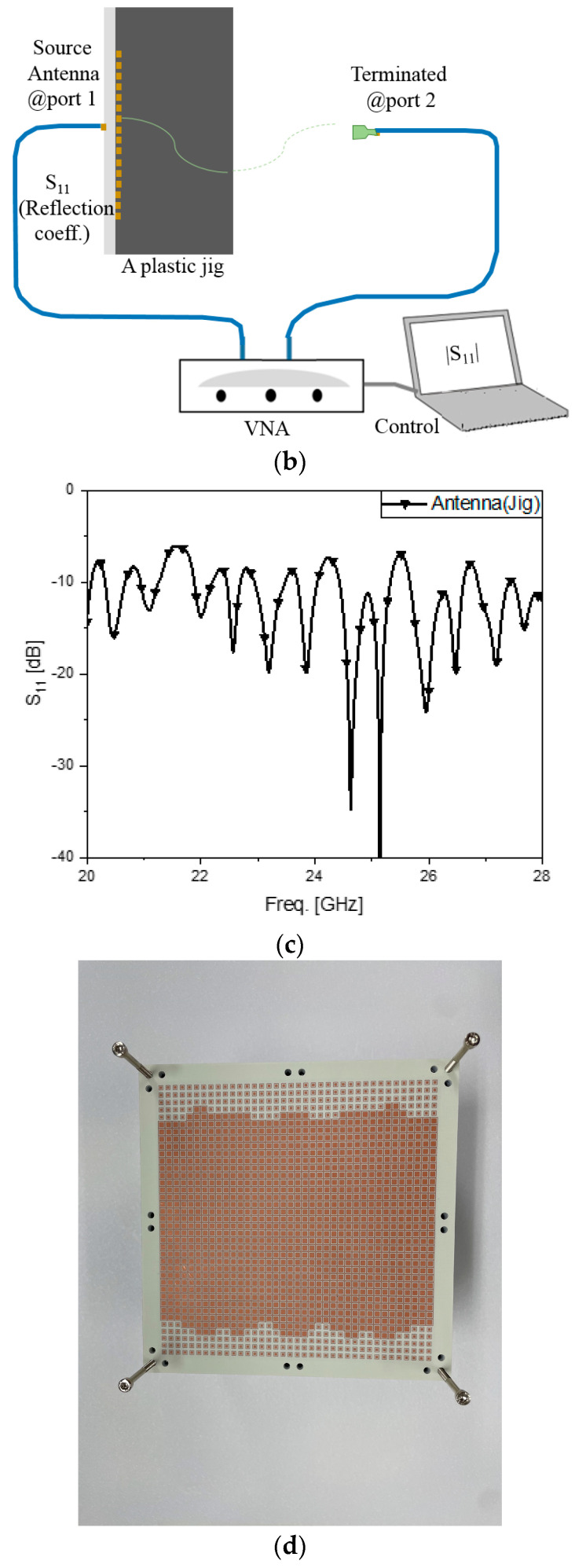

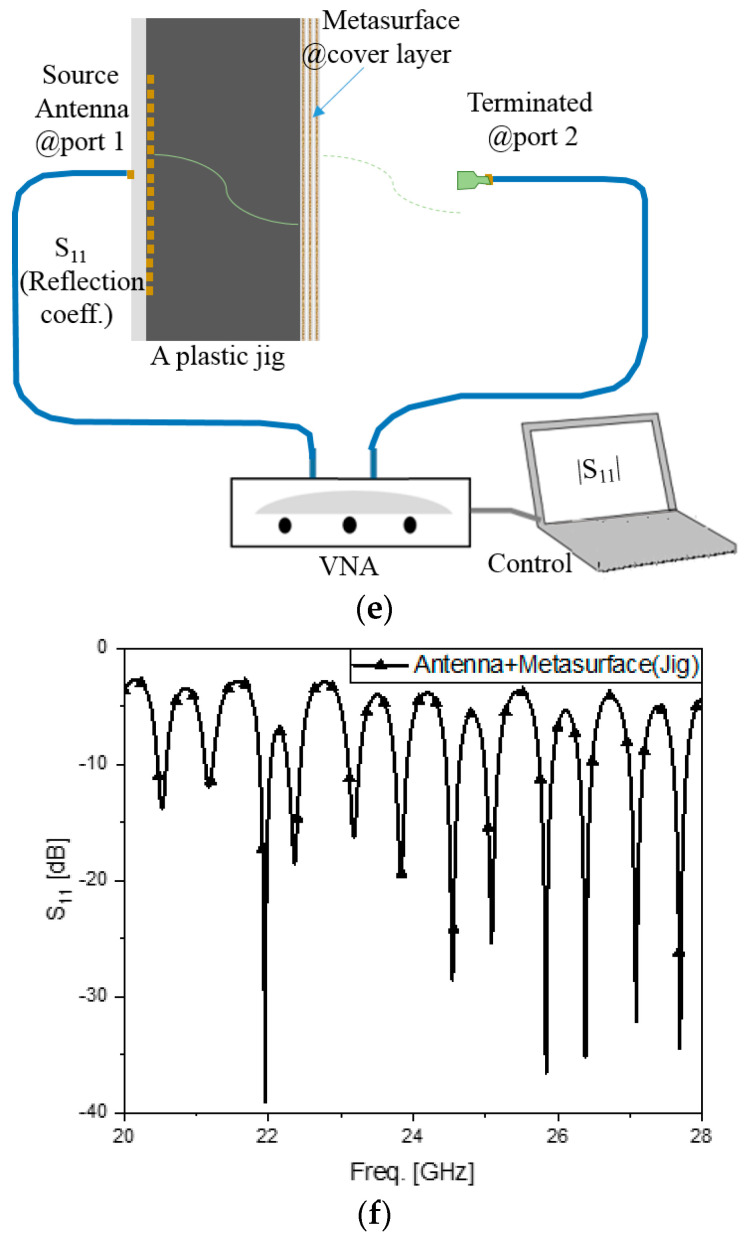
The test bench observing S_11_ of the two specimens. (**a**) The prototype of the source antenna in the jig; (**b**) schematic of measuring S_11_ of the prototyped source antenna; (**c**) measured S_11_ of the prototyped source antenna; (**d**) the prototype of the metasurface-combined antenna in the jig; (**e**) schematic of measuring S_11_ of the prototyped metasurface-loaded antenna; (**f**) measured S_11_ of the prototyped metasurface-loaded antenna.

The first specimen is the fabricated source of radiation, which is laid at the bottom layer of the 3D-printed jig as shown in Figure 3a. This antenna is excited by port 1 of the VNA while port 2 sits idle, as depicted in Figure 3b. S_11_ as the input port reflection coefficient is measured, and its result is Figure 3c. There occur many peaks, the cause of which is inferred as multiple reflection minor lobes on the antenna beam and coupling between the patches hitting the walls. As a reminder, as an array antenna is enlarged to the area made up of a great number of radiating elements, the main lobe of the beam becomes very directional and strong, but it has more and more minor lobes next to its flank. Minor lobes will be weak at the far zone, but the walls of the radar mock-up are close to them in the zone and their field intensities are not negligible. The metasurface is fabricated and mounted over the source antenna, as shown in Figure 3d. Port 1 is turned on for the antenna combined with the metasurface. The measurement referring to the configuration of Figure 3e provides S_11_ of the second specimen. Figure 3f reveals the levels of the peaks on the curve go up because the cover layer stirs the direct path from the internal medium of the jig to the external medium. Both the S11 plots have the reflection coefficients below −10 dB at 24.5 GHz. The second kind of experiment is an intuitive way of investigating the wireless link between the sides of radio sensing and its strength.

**Figure 4:**
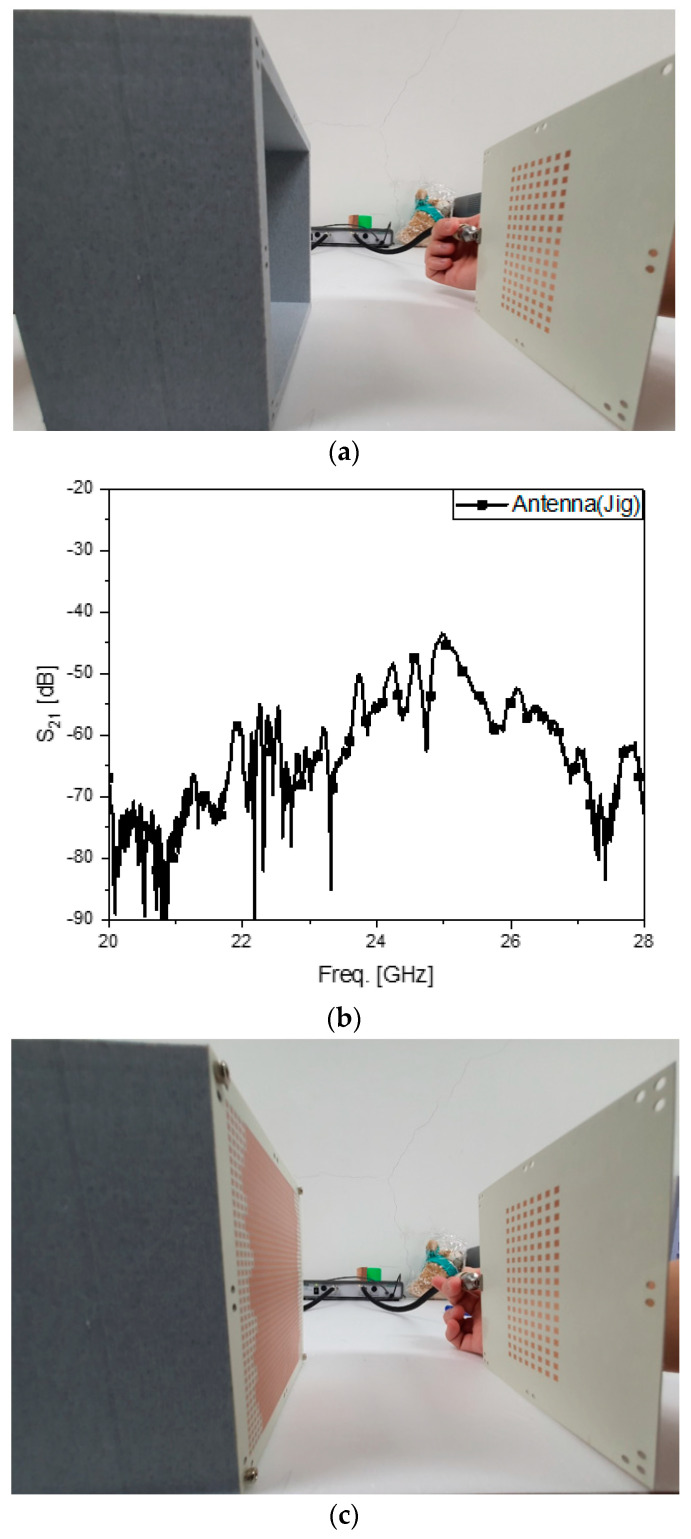

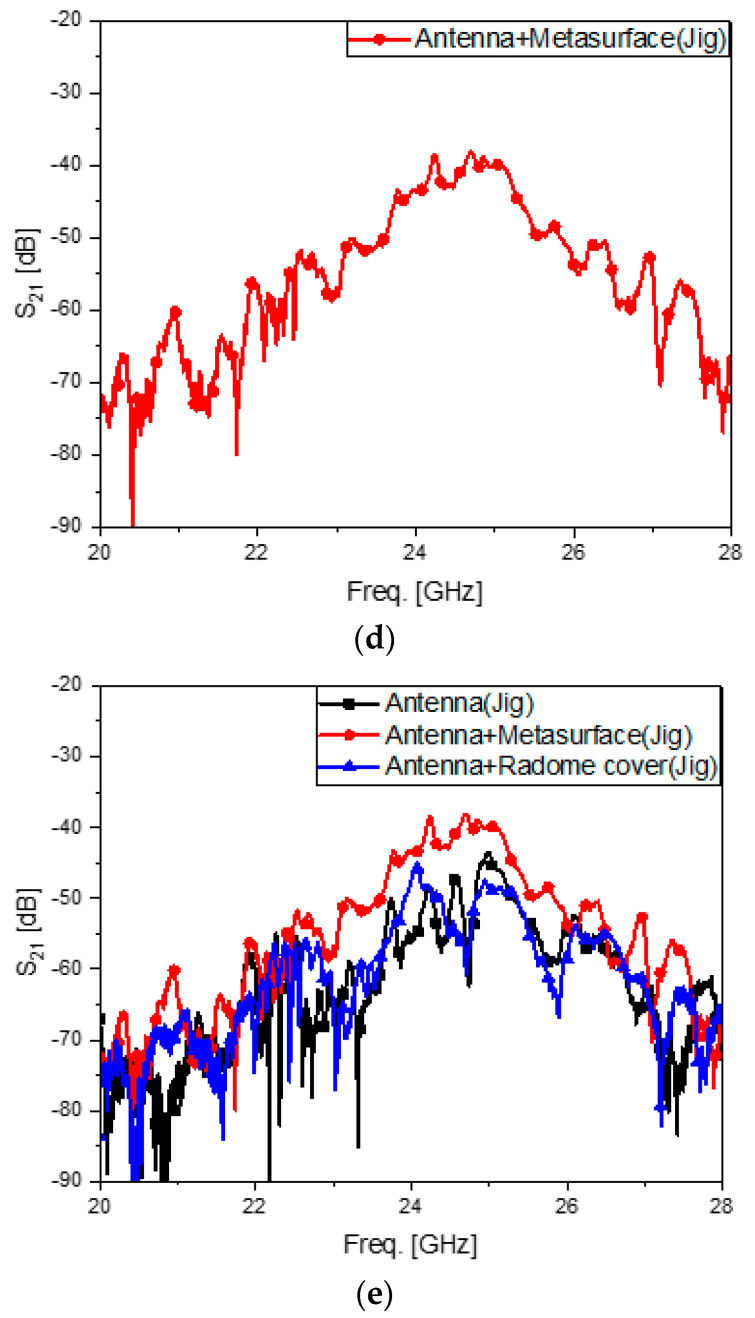
The test bench observing S_21_ of the two specimens. (**a**) Test configuration for measuring S_21_ between the twin array antennas; (**b**) measured S_21_ between the array antennas; (**c**) test configuration for measuring S_21_ between the metasurface-loaded array antenna and unloaded-array antenna; (**d**) measured S_21_ between the metasurface-loaded array antenna and unloaded-array antenna; (**e**) comparing the curves of S_21_, including a reference.

Watching the reflection coefficients of the antenna and its modified version as frequency responses is meaningful, as stated in the previous paragraph. Meanwhile, it is worthwhile to check S_21_ as the transfer coefficient between one antenna and another because it supplies quantities of electromagnetic connectivity between the two sides as useful information. Figure 4a is the test bench to observe S_21_ between the array antenna in the jig as the transmitter at port 1 and the array antenna as the receiver at port 2. It is about −47 dB at 24.5 GHz, as shown in Figure 4b. When port 1 is substituted by the metasurface-loaded antenna, as seen in Figure 4c, S_21_ reaches around −38 dB, as shown in Figure 4d, implying that using the proposed metasurface provides more than 7-dB improvement from the array without the novel cover layer. In Figure 4e, while the blue curve denoting S_21_ from the source antenna with an ordinary dielectric plane 4350B located at the cover layer for port 1 disturbs the wireless link the worst with the level at about −55 dB, the proposed structure outperforms the other ones. Comparing the three plots reveals the metasurface-combined array antenna enhances the radio link by 7~17 dB. The third type of experiment takes place in the anechoic chamber, as others do in [15,16,17,18,19,20,21].

**Figure 5:**
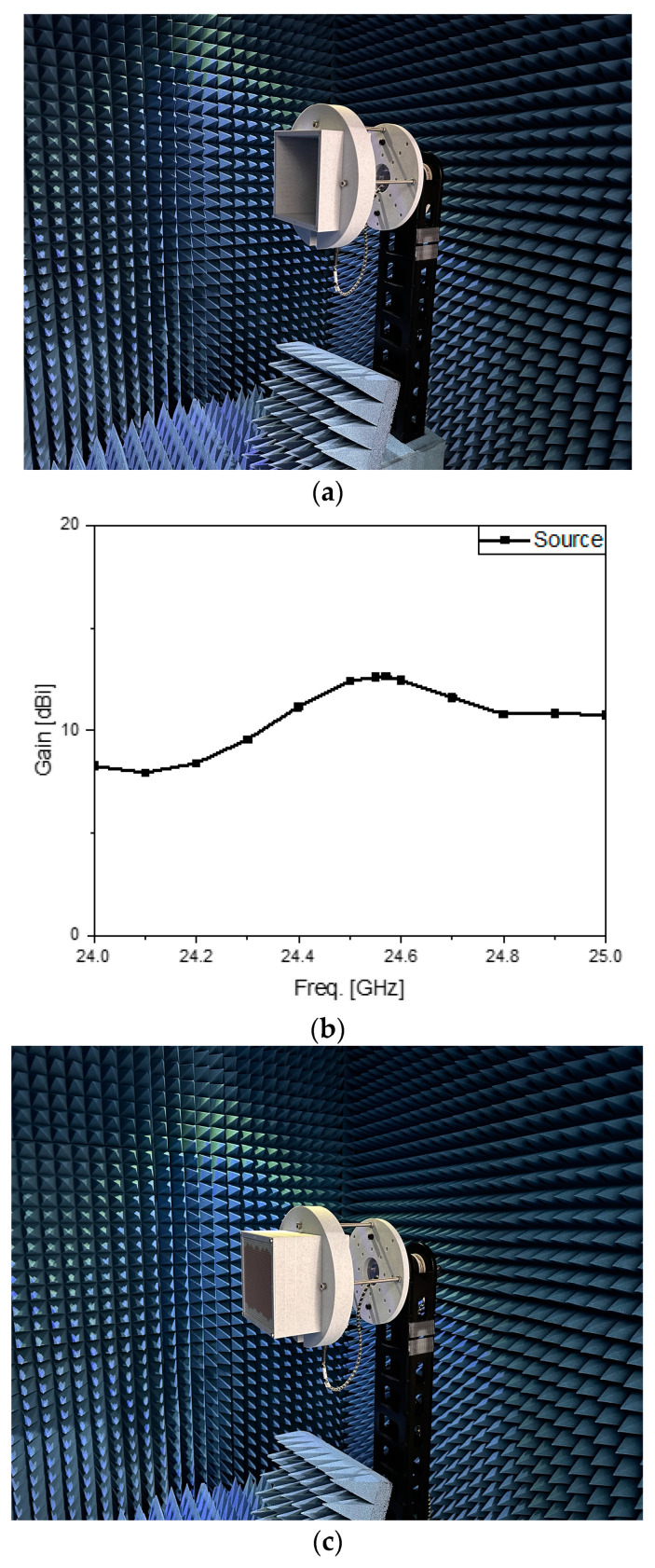

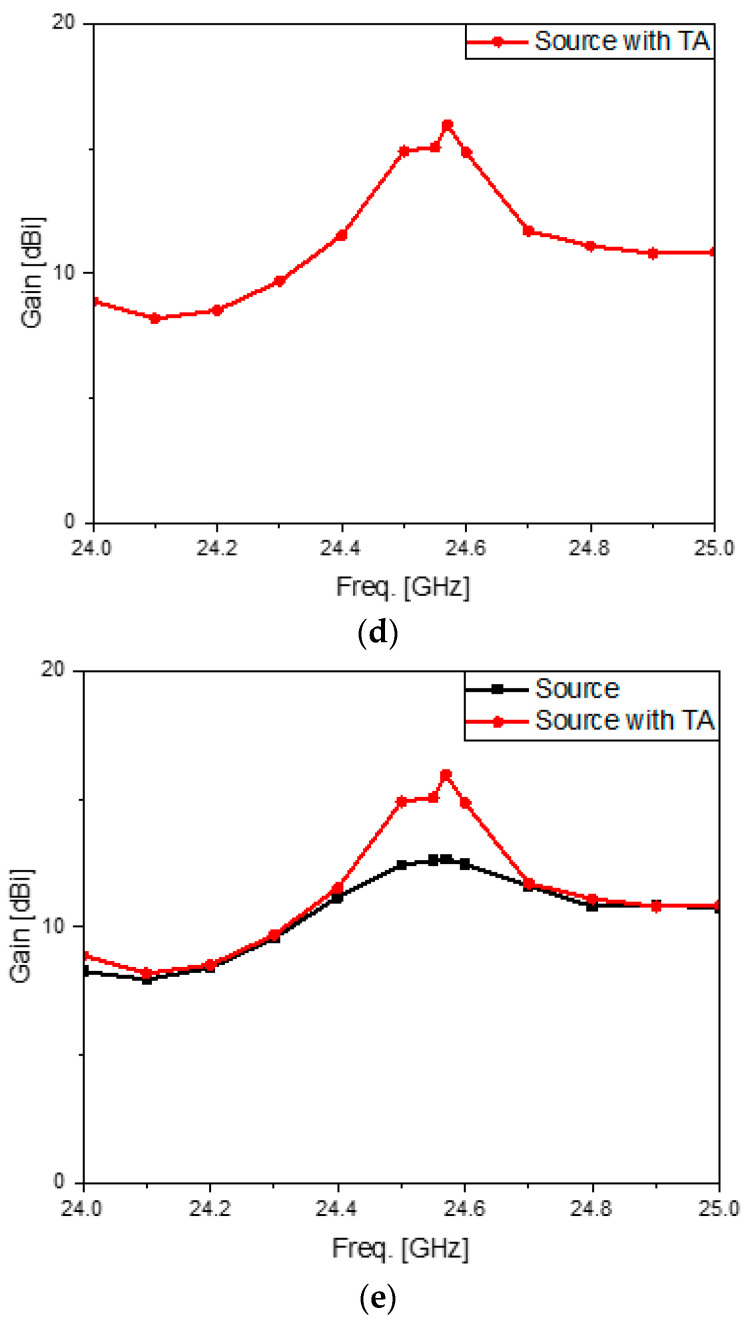
The anechoic antenna chamber tests. (**a**) Test setup for the source antenna in the jig; (**b**) measured antenna gain of the source antenna in the jig; (**c**) test setup for the metasurface-loaded antenna in the jig; (**d**) measured antenna gain of the metasurface-loaded antenna in the jig; (**e**) comparing the curves of antenna gain.

The two specimens were taken to the anechoic chamber to watch how the far fields behave. Figure 5a has the array antenna contained in the jig as the AUT. Its antenna gain is plotted in Figure 5b. Saving the data, the experiment moves on to Figure 5c, where the metamaterial-mounted source antenna is held in the fixture. The antenna gain rises to 16 dBi and the comparison presents around 5 dB improvement in Figure 5d,e. Despite errors guessed to result from the mechanical stress to the connector and cable with the turntable, the proposed structure can give advantages to a radar system.

**Figure 6:**
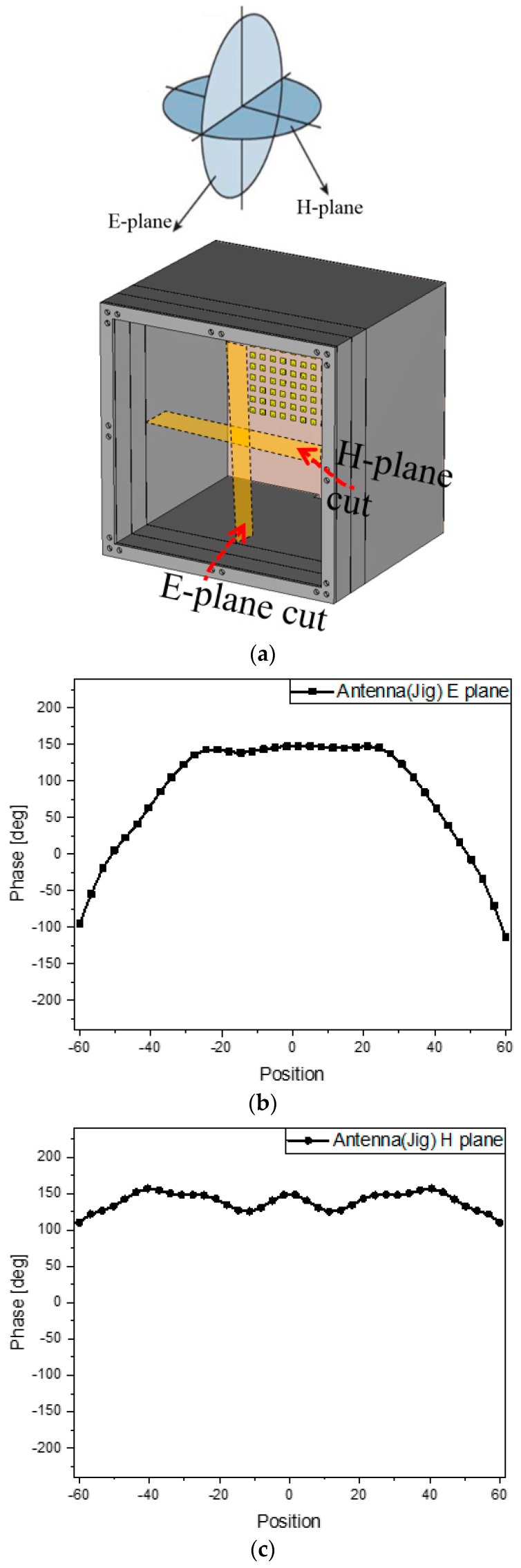

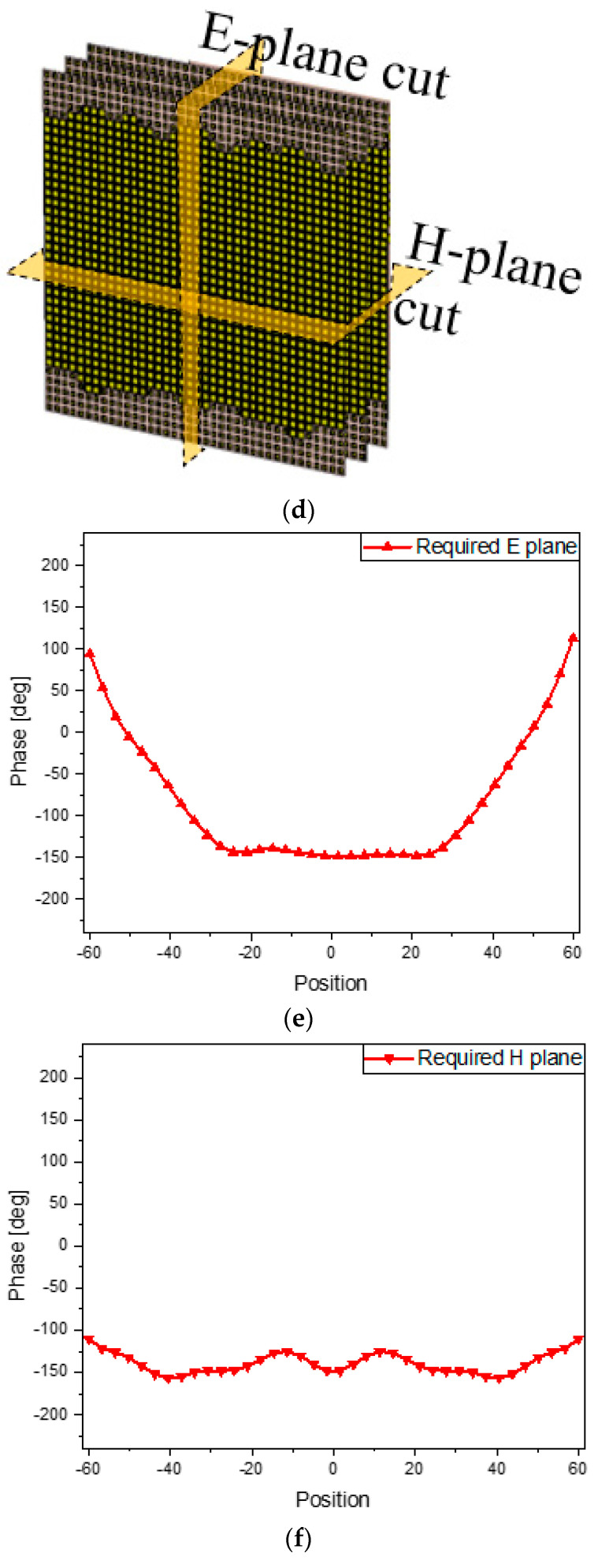

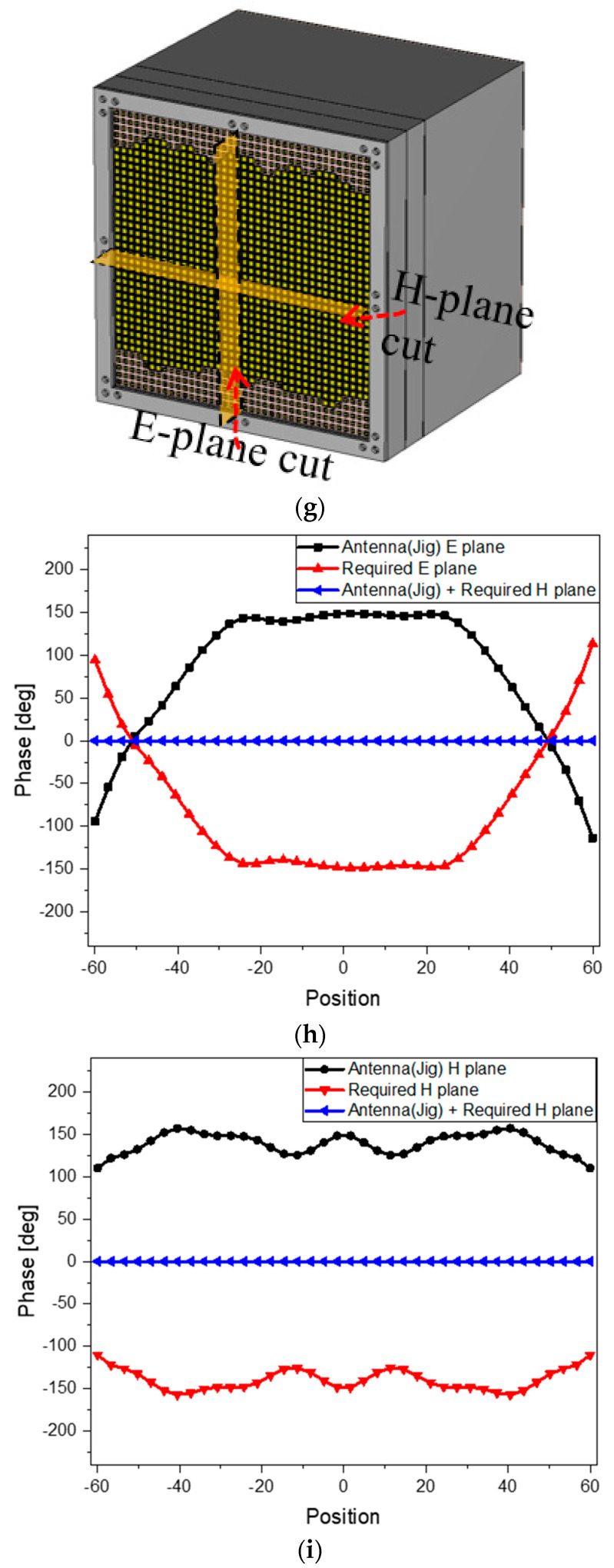
The analysis of phase maps from the steps to build the antennas. (**a**) Observation planes on the source antenna in the jig; (**b**) E-plane phase profile of the source antenna in the jig; (**c**) H-plane phase profile of the source antenna in the jig; (**d**) observation planes only on the metasurface; (**e**) E-plane phase profile of the metasurface alone; (**f**) H-plane phase profile of the metasurface alone; (**g**) observation planes on the metasurface-loaded antenna in the jig; (**h**) E-plane phase profile of the metasurface-loaded antenna; (**i**) H-plane phase profile of the metasurface-loaded antenna.

As for theoretical understanding, the way the metasurface compensates for the input phases is analyzed. Figure 6a defines the two principal planes surrounding the electromagnetic geometry. The source antenna generates the incident wave as the input to the bottom of the metasurface. The phase distribution of the input is observed on the E-plane cut and H-plane cut plotted as Figure 6b,c. The E-plane phase profile changes more rapidly going near the edges than the H-plane phase profile because the wave patterns on the E-plane and H-plane come from eight and sixteen elements, respectively. Under the goal of transmitting the plane wave or a similar one, ultimately, the metasurface alone, as shown in Figure 6d, should have the complementary phase profiles concerned with the inputs on the E- and H-planes. The phase maps required only for the metasurface are presented in Figure 6e,f. Then, combining the metasurface with the source antenna, as shown in Figure 6g, is equivalent to adding the phase map of the metasurface to the input phase map of the source antenna. The compensated phase distributions on the two principal planes appear as the blue curves in Figure 6h,i. The resultant phase or the output phase is flat. This is a proof of the desired effect.

Lastly, by examining the difference between phases of measured S_21_, the refractive index is analyzed. For a note, the refractive index is also an indicator of metamaterials.

**Figure 7:**
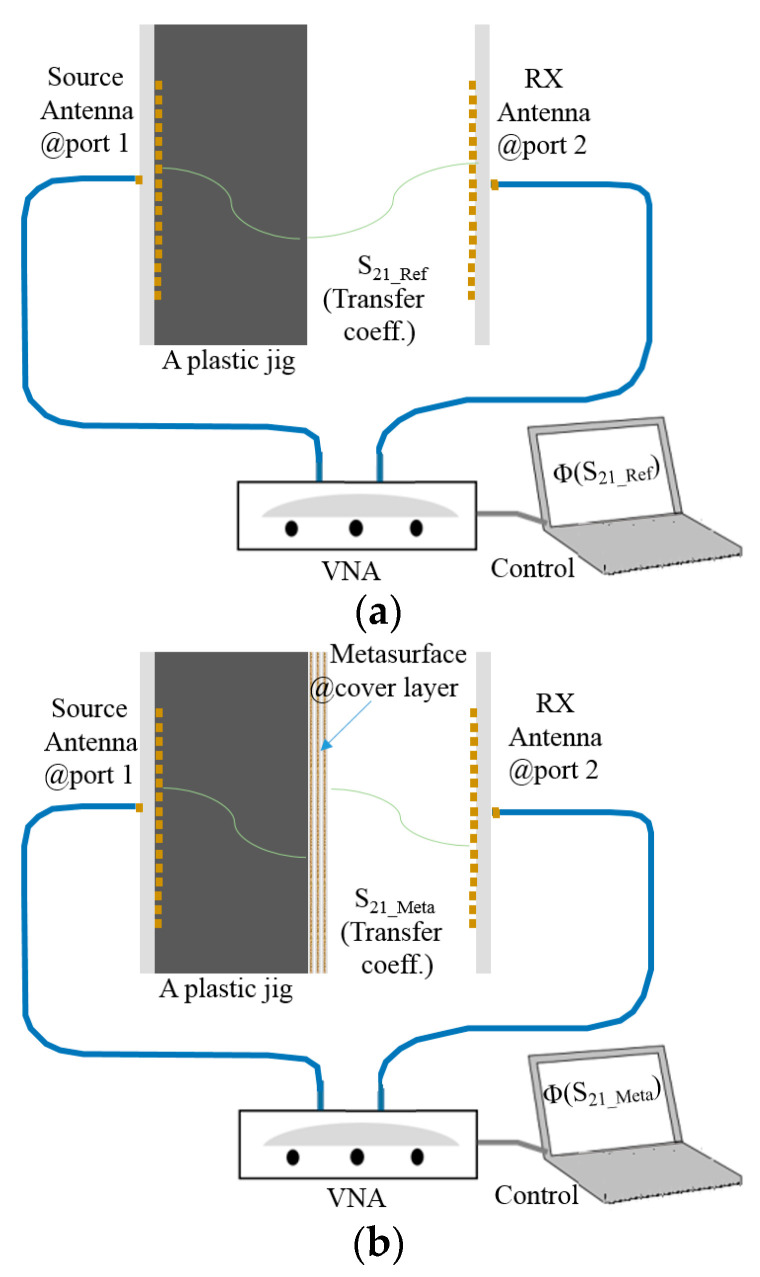

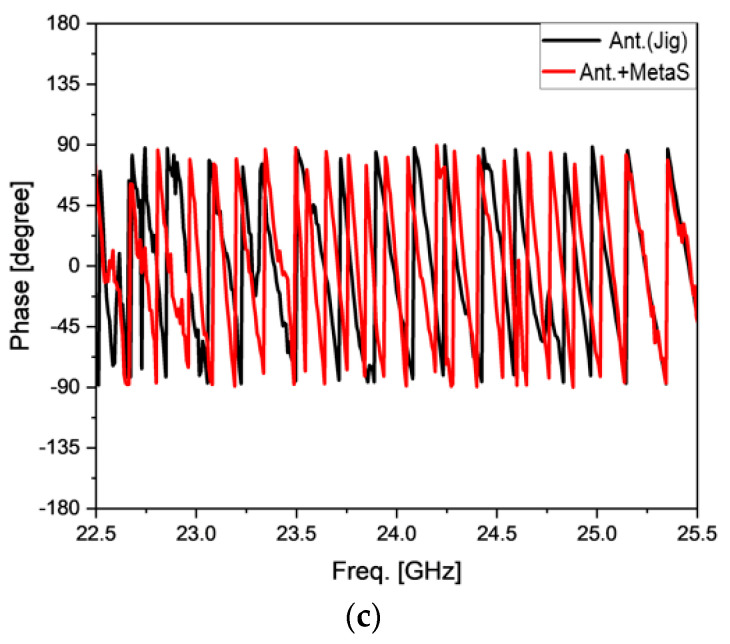
The analysis of the refractive index of the wireless link. (**a**) Test setup of viewing S_21_ without the metamaterial; (**b**) test setup of viewing S_21_ with the metamaterial; (**c**) comparing the phases of the two cases.

Electromagnetics and transmission-line theories address the relationship of the refractive index of the wave with the phase as the signature of the wireless signal traveling a certain distance in a medium. This is mathematically expressed as follows:

(1) $$∆\Phi_{i}=\beta_{0}\times \xi \times \lambda_{0}\times n_{∆}=\xi \times 2\pi \times n_{∆}$$

(2) $$n_{∆}=\frac{1}{\xi }\times \frac{∆\Phi_{i}}{2\pi }$$

where $n_{∆}$ , $∆\Phi_{i}$ , $\beta_{0}$ , $\;\lambda_{0}$ , and ξ denote refractive index, phase difference, free-space propagation constant, free-space wavelength, and ratio of distance to $\lambda_{0}$ in that order. S_21_ is obtained from configurations in Figure 7a for the TX antenna without the metasurface and Figure 7b for the TX antenna with the metasurface. The phase of S_21_ in Figure 7a is subtracted from the phase of S_21_ in Figure 7b, and this phase difference is put into Equations (1) and (2). From the comparison of the measured phases of the source antenna case and the metasurface-combined antenna case in Figure 7c, the phase changes from −69.11° to −3.87° as the phase lead, and the phase difference is 65.24°. Considering the thickness of the metasurface now, with reference to frequency 24.5 GHz for $\lambda_{0}$ = 1.21 cm with ξ = 2.5, refractive index $n_{∆}$ becomes approximately 0.072. It was found out that the metasurface enables the radiated wave of the antenna structure to behave as the plane wave with nearly zero angle of refraction.

## 4. Discussion

The crux of the proposed work was elaborated upon in the previous sections. But some of the terminologies, definitions, technical contributions, and differences, mathematical basics in design, and merits of the structure need more explanation. First, readers might be confused between metamaterials and metasurfaces and want to know which kind the proposed structure belongs to. By checking references from 9 to 21 and additional references numbered from 22 to 26, both the words are used in a mixed way. In the early days, researchers had a tendency to use metamaterials and metamaterial surfaces, but quite recently, those who design metamaterial antennas or metamaterial devices with dielectric substrates and stack them started calling their metamaterials metasurfaces, which may sound fancy. The structure proposed in this paper is thought of as a metasurface. And this definition is supported by what C. L. Holloway et al. with [22], Y. Lee et al. with [23], and A. Ali et al. with [24] said. Similar points of view are given to the antennas designed by T. J. Cui with [25] and M. E. Badawe with [26], where metasurfaces are 2D versions of metamaterials. Secondly, novelties of the proposed structure are explained by comparing the present one with other designs, which are presented in the table below.

As written in Table 2, the proposed structure has novelties in terms of the application as well as the type of source and the operational frequency compared to samples of other approaches. When the unit-cells of the metal patches loaded with chip L and C are periodically arranged in 1D and meet the condition of negative propagation constants, leak-waves in the backward region are generated and look very broad in the transverse directions. These are connected to the coaxial cable. But, lens antennas are excited by a horn or one-patch antenna in other cases. Different from them, the proposed structure has an array antenna that gives designers difficulty in calculating and placing the phase distribution on the metasurface, which was overcome and adopted to the replacement of the ordinary radome. The array antenna as the source of radiation was designed based on the following equations to make each of the elements resonate at the millimeter-wave frequency.

(3) $$f_{Res}=\frac{1}{2\times L_{Patch}}\times \frac{v_{0}}{\sqrt{\varepsilon_{r}}}$$

(4) $$W_{Patch}=\frac{v_{0}}{2\times f_{Res}}\times \frac{\sqrt{2}}{\sqrt{\varepsilon_{r}}}$$

(5) $$E_{PropArray}(r)=E_{PropPatch}(r)\times \sum_{m=1}^{M}\left(I_{m}e^{-j\Phi_{m}}\right)\times \sum_{n=1}^{N}\left(I_{n}e^{-j\Phi_{n}}\right)$$

The unit element of the array antenna is a patch and, for the resonance frequency, *L_Patch_* the length of the patch and *W_Patch_* the width are determined by Equations (3) and (4). This resonance causes the element to propagate as $E_{PropPatch}(r)$ the electric field at ***r*** an observation position and results in $E_{PropArray}(r)$ the propagating electric field of the array antenna interpreted as the product of the element factor and the array factor comprising phase differences $\Phi_{m}$ and $\Phi_{n}$ in the *x*-axis with amplitude $I_{m}$ and *y*-axis with amplitude $I_{n}$ , respectively. Finally, the advantages are mentioned by a comparative study. The metasurface is replaced by a realistic radome or a radome with wet tissue, which mimics a worse case of higher attenuation by precipitation. In the first place, the radome bought from the market is attached to the end of the jig and its transmission coefficient is −55 dB. Compared to that of the source antenna, it is lower by 7 dB as the insertion loss. The use of the metasurface increases the signal strength by 17 dB from the ordinary radome. Going further, to model precipitation that makes the signal at the millimeter-wave very weak, a sheet of tissue is made wet by a water spray and attached to the realistic radome. This causes 7 dB to 10 dB degradation in the signal strength. This implies care must be taken of that because attenuation by precipitation is severe for K- and Ka-band antenna fields, and water-repelling agents are painted on the surface of the radome.

## 5. Conclusions

As the radar system is required to operate with accuracy and fine resolution, it is an effective way to make the antenna work suitably by beating the odds. A metasurface is suggested to overcome the shortcomings of the millimeter-wave antenna when confronting the cover layer as the radome of the system. Since the dielectric loss of the radome worsens the function of the antenna, which already undergoes path loss in the 24 GHz-band radio, the radome is replaced by a phase-manipulating surface, preventing the cover layer from disturbing the incoming wave of the source antenna. The array antenna as the source of radiation and its metasurface-incorporated version were designed and physically realized. The improvement by the proposed structure was checked by electromagnetic simulations and validated by a variety of the experiments. The test bench examination and the far-field tests revealed that the metasurface brings about 5 dB and 7 dB jumps in signal strengths. Furthermore, analyses were conducted to show valid procedural steps to achieve the constant phase distribution at the outermost aperture of the radar as a key to the high directivity of the beam imitating the plane wave and prove the measured refractive index almost zero, which was attained with the metamaterial structure.

## Figures and Tables

**Table 1:** Physical dimensions of the 180°- and 0°-pixels.

Parameter	Value
W_Lp_	0.1 mm
L_Px_	3.5 mm
g_in_	0.2 mm
L_Lp_	2.0 mm for pixel 180° (3.1 mm for 0°)
L_Rc_	1.1 mm for pixel 180° (2.1 mm for 0°)

**Table 2:** Comparison between the proposed and other methods.

	Unit-Cell Geometry	Entire Geometry	Source Type	Frequency Band	Application
[12]	Planar	2D (Layered)	Horn	Ku-band	High-gain
[16]	Planar with chip L & C	1D (Chain of cells)	Coax to the TX-line	UHF-band	Leakywave
[21]	Planar with chip L & C	1D (Chain of cells)	Coax to the TX-line	UHF-band	Broad-beam
[22]	Sphere	2D (1 plane)	Horn	UHF-band	Surface wave
[26]	Planar	2D (Layered)	Horn	UHF-band	High-gain
This work	Planar	2D (Layered)	Array	K-band (mm-Wave)	Radome

## Data Availability

Data are contained within the article.
